# Cone-Beam Computed Tomography (CBCT) Analysis of an Unusual Configuration of the Upper First Molar With a C-shaped Canal With Apically Fused Roots: A Case Report

**DOI:** 10.7759/cureus.36474

**Published:** 2023-03-21

**Authors:** Kapil D Wahane, Anand V Bansod, Sudha mattigatti, Rushikesh Mahaparale, Yuvraj B Rote, Mayur B Wanjari

**Affiliations:** 1 Department of Conservative Dentistry and Endodontics, School of Dental Sciences, Krishna Institute of Medical Sciences Deemed to Be University (KIMSDU), Karad, IND; 2 Department of Research and Development, Jawaharlal Nehru Medical College, Datta Meghe Institute of Higher Education and Research, Wardha, IND

**Keywords:** cone-beam computed tomography systems, obturation technique, maxillary molars, mandibular molar, c-shaped canal

## Abstract

Lack of identification of the root canal results in root canal treatment failure, one of the most frequent causes of root canal treatment failure. To successfully treat root canals, it is crucial to have a detailed understanding of root canal configuration, including distinguishing traits and anatomical differences. The root canal in the C form arrangement is among the most important anatomical variances. Because of its distinctive highlight, the existence of fins or webs linking the different root canals - the C-shaped form of root canal has proven challenging to detect and manage. Any molar region may have this root canal arrangement, including the mandibular first molar, first^ ^premolar, and maxillary molars. Above all, mandibular second molars are where it is most usually discovered. This report discusses the uncommon maxillary first molar with an apically merged root. The importance of comprehending canal variations, which are C-shaped root canals, should be critically evaluated in light of the rise in the root canal treatment failure rate for the upper molars.

## Introduction

There are endodontic and anthropologic implications to the study of root and canal anatomy. Understanding the differences in tooth structure and distinguishing characteristics across different ethnic groups is crucial as it may help recognize and treat those canals. The canal system's *C* design is one of the most significant anatomical variances. The C-shaped canal is due to the cross-sectional architecture of the root and canal morphology, initially described in endodontic literature by Cooke and Cox in 1979 [1-3]. Hess and Zürcher noted the intricacy of the maxillary molars' root canal system back in 1925 [4].

Probably, the tooth with the widest range of anatomical differences is the maxillary first molar. There have been occurrences of aberrant root counts, morphological abnormalities, and C-shaped root canals in maxillary first molars documented in the past [5]. As a result, the clinician must carefully inspect the maxillary molars' root canal system, preferably using a surgical operating microscope's magnification [5]. Conventional radiographs with proper angulation are of little utility in identifying the intricacy of C-shaped canal patterns due to their intrinsic limitations. The root canal structure of the tooth can be fully understood through the use of cone-beam computed tomography (CBCT) systems, three-dimensional (3D) reconstructions, and spiral computed tomography [6-8]. The first molar with a C-shaped incidence was 1.1%, and the second molar prevalence was 3.8% globally [9].

This case study shows an uncommon root canal architecture of the first molar in the maxillary arch with apically joined roots and C-shaped canal morphology. This root canal anatomy was initially detected radiographically and was later verified by CBCT systems.

## Case presentation

**Figure 1:**
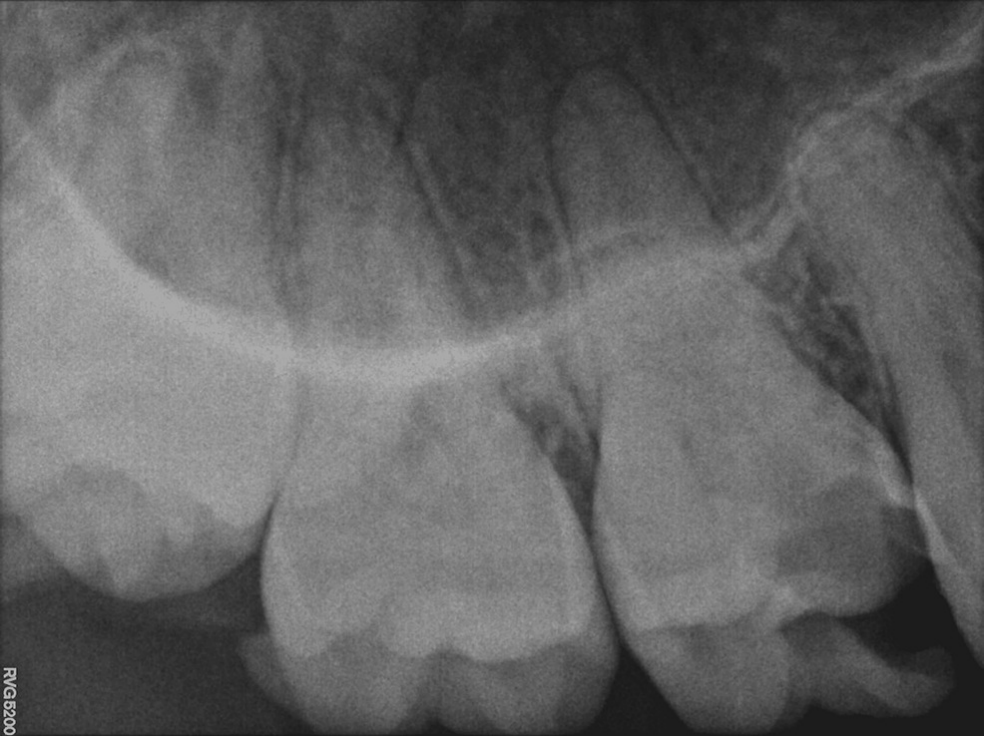
Preoperative radiograph.

A 29-year-old female patient reported to the Department of Endodontics and Conservative Dentistry with discomfort in the upper left rear area of the jaw as her main complaint. Mesial caries on the maxillary first molar were discovered during the investigation. The pain was sharp and continuous, present with nocturnal pain. Deep cavities with pulp involvement and alterations in the periapical area were seen upon radiographic inspection. Although in the preoperative radiograph, the root canal anatomy appeared unusual, the radiograph, being two-dimensional, was not confirmatory about the type of variation (Figure 1). Therefore, the patient refers for CBCT imaging.

**Figure 2:**
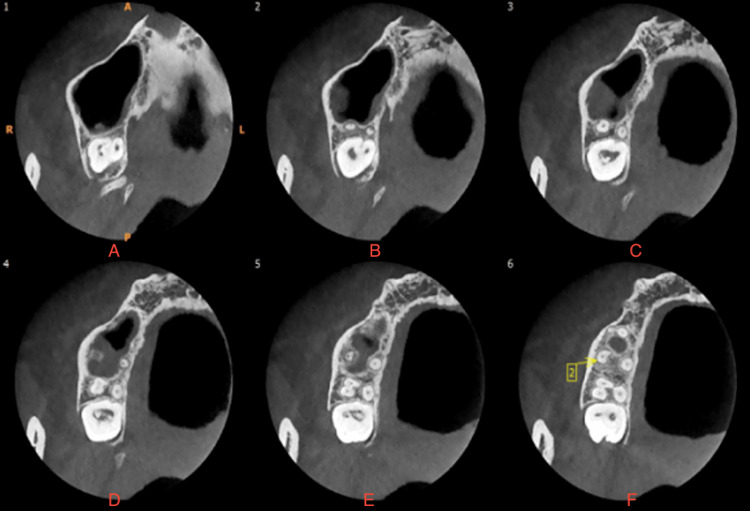
CBCT systems transverse section of teeth: (A-F) CBCT images of different levels from the apical direction; 2: a single apical opening of the root canal after fusion of the mesiobuccal and distobuccal roots. CBCT, cone-beam computed tomography

**Figure 3:**
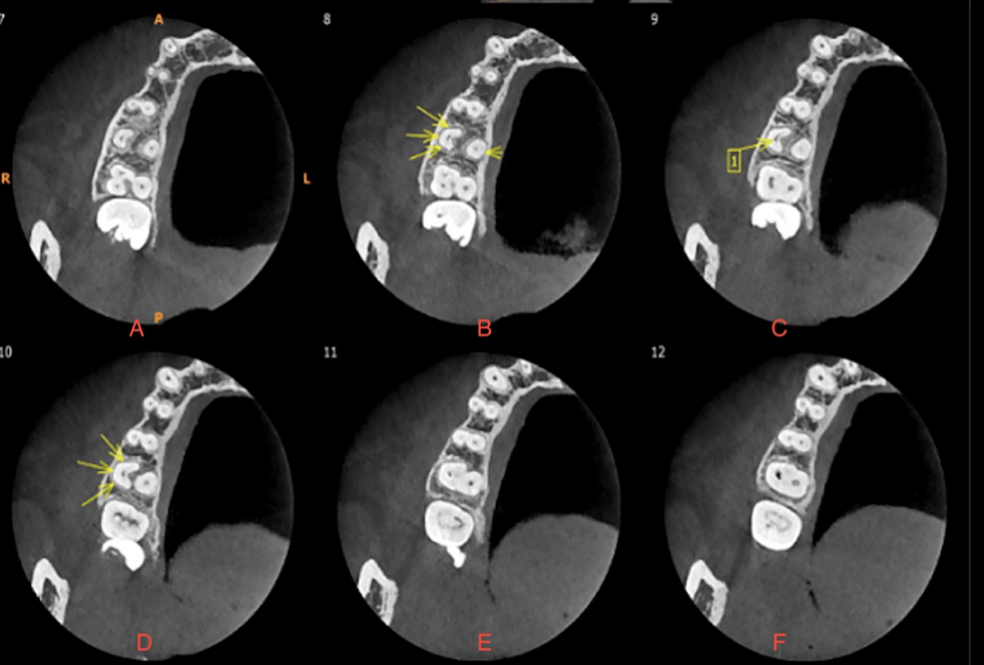
CBCT of the transverse section of teeth: (A-F) CBCT images at different levels coronally; 1: C-shaped root canal after fusion of the mesiobuccal and distobuccal roots. CBCT, cone-beam computed tomography

The patient was referred for a CBCT scan, confirming the unusual anatomical variation. On CBCT reports, it was observed as a C-shaped configuration and merging of the mesiobuccal and distobuccal canals, which were joining apically, forming only one canal (Figures 2-3). The palatal canal was separate and patent. Clinical and radiological examination confirmed the diagnosis of acute irreversible pulpitis concomitant with apical periodontitis. Then root canal treatment was advised, and consent was taken. The access cavity was prepared.

**Figure 4:**
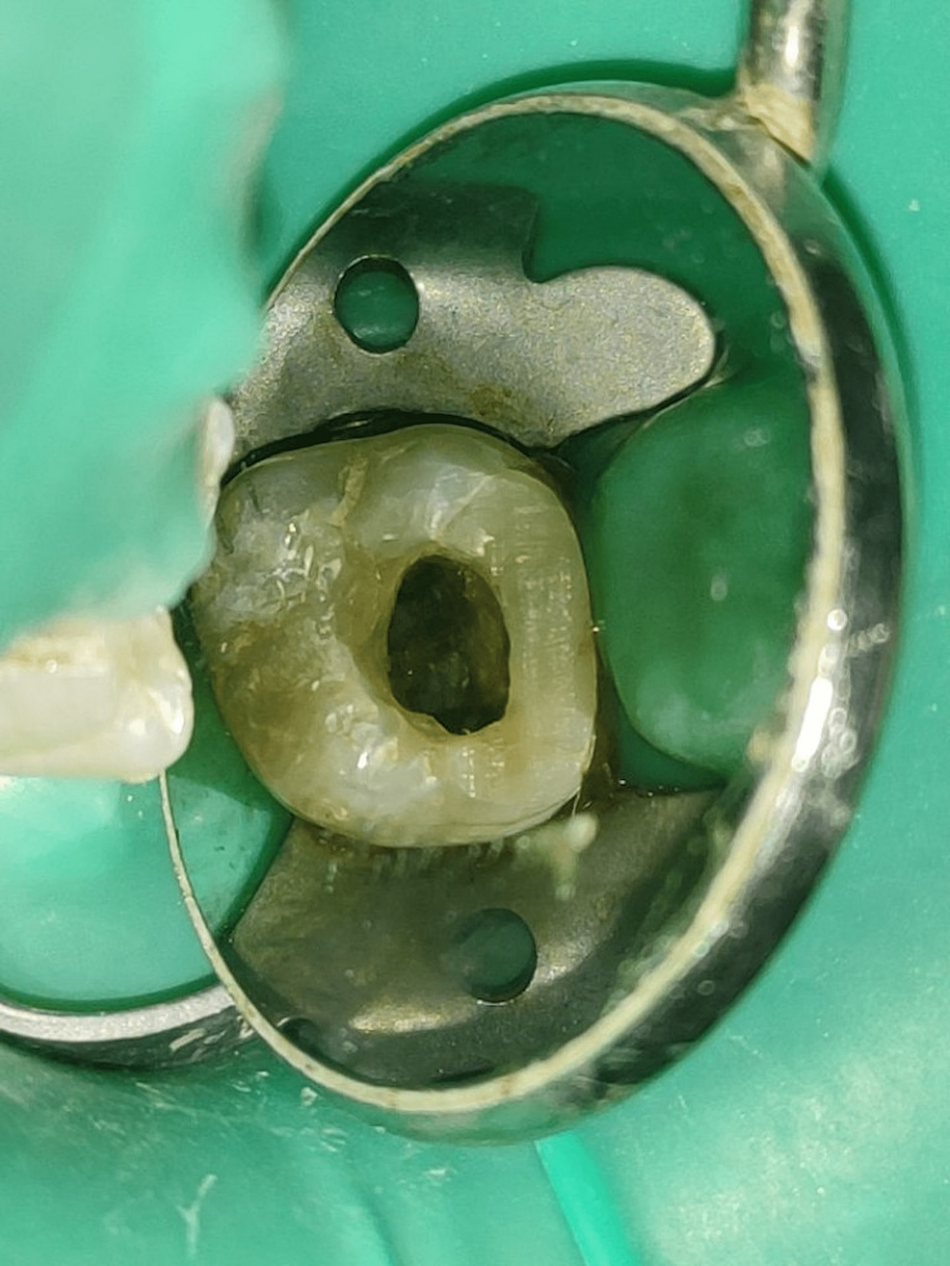
Clinical photograph of the access opening.

After giving local anesthesia to the area, a root canal access was opened with the help of a Tulsa Endo Z bur and an Endo Access bur ((Dentsply Maillefer, Tulsa, OK, USA) with a proper sterile condition using a rubber dam. Exploration following the dentin map confirmed the existence of two root canals: C-shaped buccal and palatal (Figure 4).

**Figure 5:**
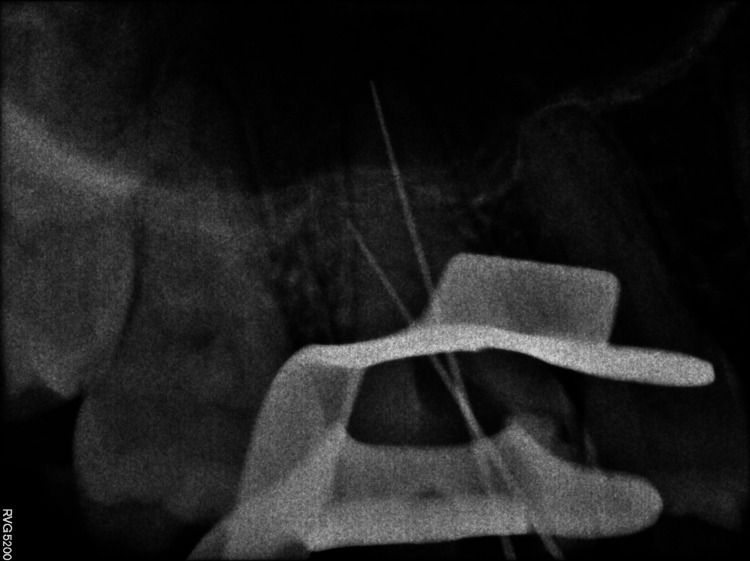
Working length determination.

The inflamed pulp was removed, and canals were properly irrigated with normal saline and 3% sodium hypochlorite. The working length (WL) was taken using the Root ZX mini apex locator (J. Morita, Tokyo, Japan) and confirmed by intraoral periapical radiographs (Figure 5).

**Figure 6:**
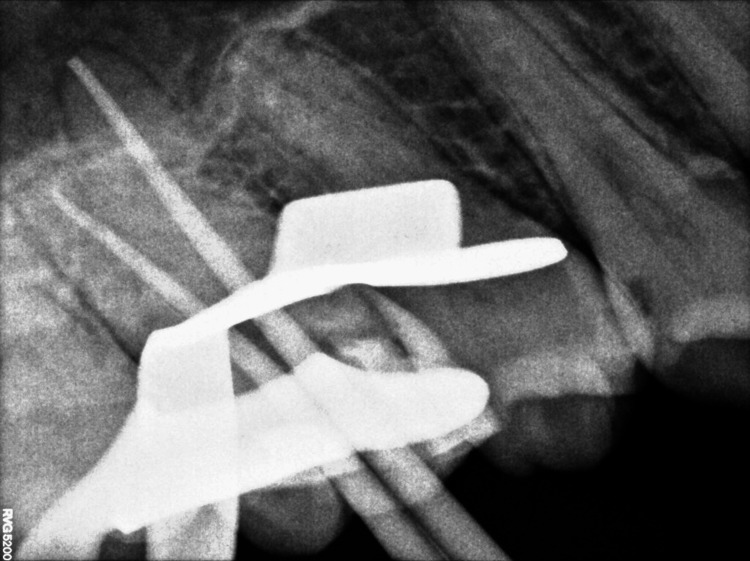
Master cone selection.

**Figure 7:**
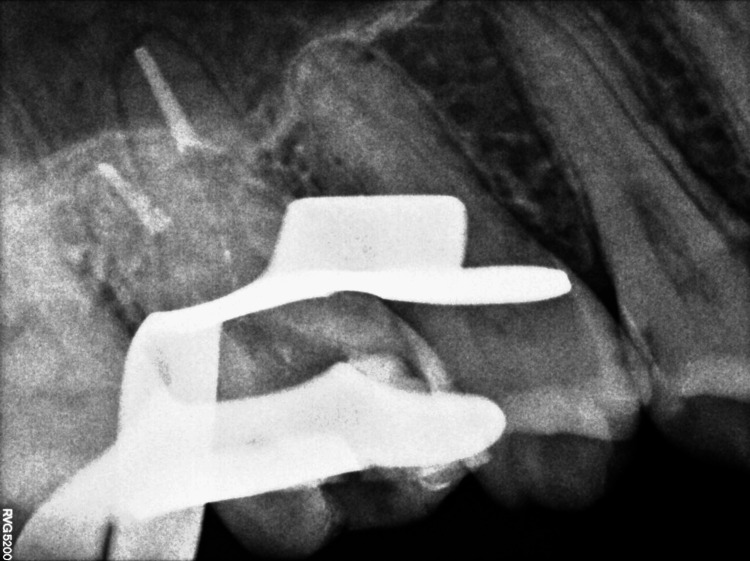
Sectional obturation.

By inserting an ISO#10 K file into the tooth, biomechanical preparation (BMP) was begun to assess the canal's patency. BMP was completed by sequentially using nickel-titanium ProTaper universal rotary files up to F4 (Dentsply Maillefer, Ballaigues, Switzerland). All canal walls were prepared by circumferential brushing and 3% NaOCl, and normal saline was used to irrigate the root canals. After administering an intracanal calcium hydroxide medication for one week, the patient was called back. Master cones were selected initially; sectional obturation was carried out (Figures 6-7).

**Figure 8:**
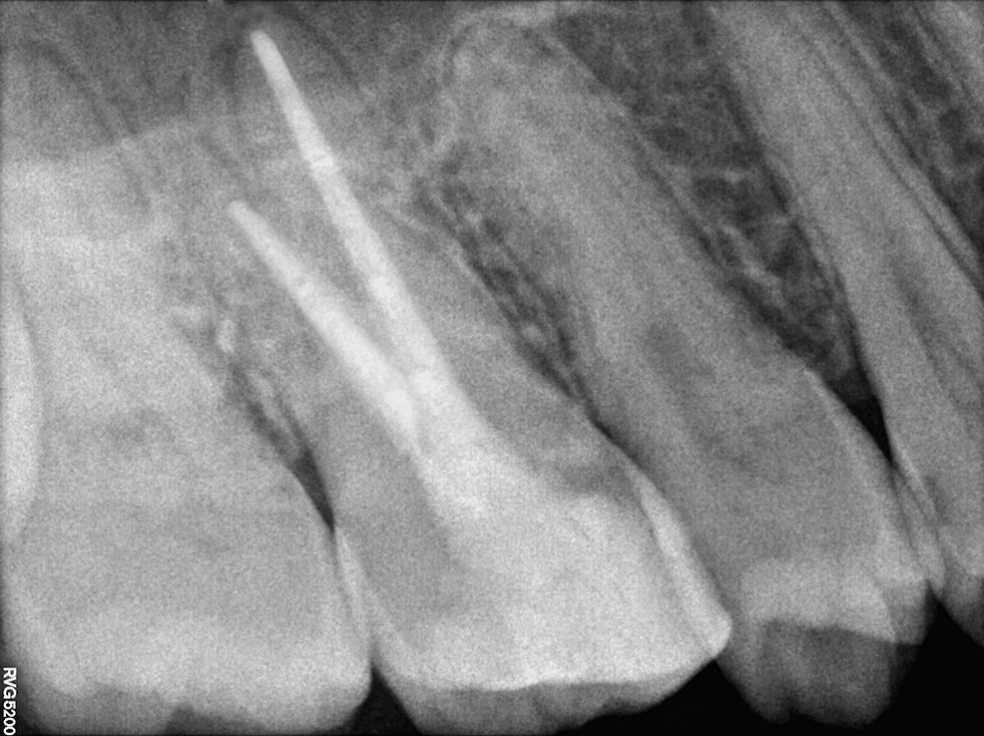
Post-obturation radiograph.

Final obturation was performed using the thermoplasticized gutta-percha obturation technique (Cordless Root Canal Obturator System B™, SyBron Endo, Chicago, IL, USA) and AH Plus (Dentsply Maillefer, Tulsa) root canal sealer (Figure 8), and the coronal restoration was completed to stop microleakage and tooth breakage.

**Figure 9:**
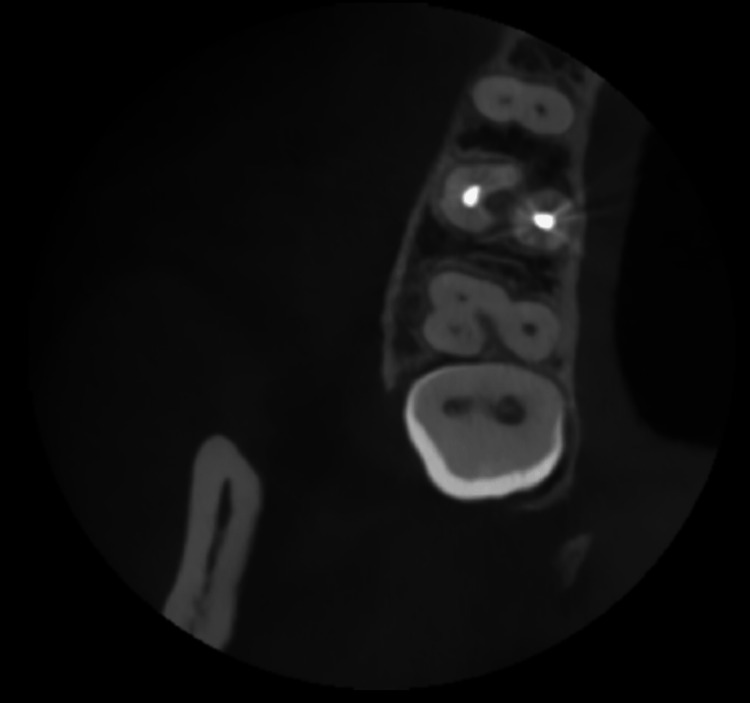
Postoperative CBCT imaging. CBCT, cone-beam computed tomography

Postoperative CBCT images were taken to confirm the 3D and uniform obturation of the prepared root canal space (Figure 9).

## Discussion

The current case has shown that the maxillary first molar possesses a C-shaped root canal morphotype, often observed in the lower jaw, that is, mandibular molars. A C-shaped root has always been theorized to result from Hertwig's epithelial root sheath (HERS) failing to fuse on the lingual or buccal root surface. The C-shaped root morphology may also develop through coalescence due to the gradual cementum deposition. It is quite uncommon to record the first molars in the maxilla having C-shaped canals, and only a few writers have done so in the past. According to De Moor's analysis in 2002, only 0.091% of maxillary first molars had C-shaped canals [5]. In 2006, Cleghorn et al. observed that just 0.12% of maxillary first molars had C-shaped roots and canals [6]. Most of the evidence in the literature comes from research (in vitro), in vivo investigations of the architecture of the clinical root canal system, or clinical cases, with two-dimensional radiographs serving as tools for verifying the accounts [7,8]. Although it can also occur in maxillary and mandibular molars, C-shaped morphology in the root canal is an anatomical variant mostly found in mandibular second molars, notably in the Asian population [9,10].

The appearance of a web linking the separate root canals is the primary anatomical characteristic of C-shaped canals. Conical or square roots are typically seen in teeth having C-shaped canals [11]. Only a few publications mention maxillary first molars with C-shaped canals [12,13]. These teeth illustrated the C-shaped canal formed when the palatal and distobuccal canals merged. According to the classification by Jo et al., the present case falls in type I fusion of two root canals and subtype C (mesiobuccal-distobuccal root canal fused) [14]. The root canal morphology in C-shaped maxillary teeth has been formed by the merging of two roots. Type I with subtype C (mesiobuccal-distobuccal root canal fused), subtype A (mesiobuccal-palatal root canal fused), and subtype B (distobuccal-palatal root canal fused) is arranged in decreasing order of incidence. Joining the three roots, that is, type II with subtype A (distobuccal-mesiobuccal-palatal root canal fused) and subtype B might result in a bigger C-shaped configuration (mesiobuccal-palatal-distobuccal root canal fused). The most prevalent types were fused roots (mesiobuccal-palatal root canal fused) and type I subtype C (mesiobuccal-distobuccal root canal fused) of a C-shaped canal [14,15].

Only two canals were found in the present case, one on the palatal side and the other on the buccal side of the access cavity. The distal and mesial canals in the buccal root combined to create a single C-shaped canal that emerged as a single apex - type I subtype C (mesiobuccal-distobuccal root canal fused) of a C-shaped canal. A single, broad palatal root canal was seen [14]. In these types of modified canals, the obturation technique becomes mandatory. The thermoplastics obturation technique was used to fill the C-shaped canal in a uniform and 3D manner. The case illustrates the significance of a comprehensive assessment of the pulp chamber's floor and a very unusual one; their significance for the prognosis of a particular patient should not be undervalued [15].

As a limitation of this study, although having a third dimension, CBCT images' spatial resolution (0.4-0.076 mm, or 1.25-6.5 line pairs per millimeter) is less than that of traditional film-based (about 20 line pairs per millimeter) or digital (varying from 8 to 20 line pairs per millimeter) intraoral radiography. The best resolution for CBCT images in endodontics is always task-specific, but most endodontic procedures need imaging of small structures [16]. The use of CBCT, which has a larger radiation dose than standard radiography, is only permitted in a small number of circumstances and only after a thorough clinical assessment [17].

## Conclusions

Early detection of canal configurations makes cleaning, shaping, and obturating a root canal easier. In this instance, we have reported on treating the upper maxillary first molar's C-shaped design. The 3D CBCT scan demonstrated a connection between exterior and interior root morphology by showing a fusion of mesial and distobuccal roots with a single apical root end. The endodontist should be aware of these root canal morphology variations in maxillary molars as it is one of the most crucial criteria for effective root canal therapy.
